# Potential nanotherapeutic strategies for perioperative stroke

**DOI:** 10.1111/cns.13819

**Published:** 2022-03-04

**Authors:** Jingyi An, Ling Zhao, Ranran Duan, Ke Sun, Wenxin Lu, Jiali Yang, Yan Liang, Junjie Liu, Zhenzhong Zhang, Li Li, Jinjin Shi

**Affiliations:** ^1^ School of Pharmaceutical Sciences Zhengzhou University Zhengzhou China; ^2^ Key Laboratory of Targeting Therapy and Diagnosis for Critical Diseases Zhengzhou China; ^3^ Key Laboratories of the Ministry of Education Zhengzhou University Zhengzhou China; ^4^ Department of Neurology The First Affiliated Hospital of Zhengzhou University Zhengzhou China; ^5^ Department of Urinary Surgery The First Affiliated Hospital of Zhengzhou University Zhengzhou China; ^6^ Department of Anesthesiology Beijing Friendship Hospital Capital Medical University Beijing China

**Keywords:** anti‐inflammatory, antioxidant, nanodelivery system, neuronal regeneration, perioperative stroke, thrombolysis

## Abstract

**Aims:**

Based on the complex pathological environment of perioperative stroke, the development of targeted therapeutic strategies is important to control the development of perioperative stroke.

**Discussions:**

Recently, great progress has been made in nanotechnology, and nanodrug delivery systems have been developed for the treatment of ischemic stroke.

**Conclusion:**

In this review, the pathological processes and mechanisms of ischemic stroke during perioperative stroke onset were systematically sorted. As a potential treatment strategy for perioperative stroke, the review also summarizes the multifunctional nanodelivery systems based on ischemic stroke, thus providing insight into the nanotherapeutic strategies for perioperative stroke.

## Graphical abstract

Potential nanotherapeutic strategies for perioperative stroke. Intravenous thrombolytic strategies include extending the “therapeutic window,” improving the effectiveness of tPA thrombolysis, and improving targeting; the antioxidant strategy includes delivery of natural antioxidants, improving the efficiency of BBB delivery, and nanoenzymes; the anti‐inflammatory strategy includes gene silencing and reducing inflammatory cell infiltration; and the neuronal regeneration strategy includes delivery of neurotrophic reagents and stimulation of trophic factor regeneration.

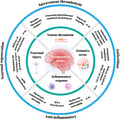

With the advent of “global aging,” stroke has become the world's second‐deadliest disease after coronary heart disease, accounting for 10%–15% of global deaths.^1^, ^2^, ^3^ The perioperative period can be a high‐risk period for stroke because of the pathophysiological state of the patient, which is based on the disease, anesthesia, functional changes in the coagulation system and pharmacological factors.^4^, ^5^, ^6^ Recently, the number of patients with perioperative acute ischemic stroke (PAIS) has increased significantly.^7^ Regrettably, clinical approval for the prevention and treatment of perioperative ischemic stroke has not yet been obtained.^8^ Therefore, the development of more therapeutic strategies for perioperative stroke is urgently needed.^9^, ^10^ Given the similar pathophysiology of perioperative strokes and simple stroke, nanotherapeutic strategies for simple stroke are also potential for the treatment of perioperative stroke. Therefore, the current nanotherapeutic strategies for simple stroke are summarized to provide insight into the nanotherapeutic strategies for perioperative stroke.

## 1 DEFINITION AND CLASSIFICATION OF PERIOPERATIVE STROKE

The World Health Organization (WHO) defines perioperative stroke as a focal or diffuse cerebral neurological deficit caused by intraoperative or postoperative cerebrovascular etiology, which can last up to 24 h or result in death within 24 h of occurrence. Based on perioperative stroke data, hemorrhagic strokes account for 1%–4% of strokes, and perioperative strokes are predominantly embolic.^11^ The mechanism of embolism in perioperative stroke is not well understood and may be related to the following factors (Table 1).

**TABLE 1:** Possible mechanisms for the occurrence of ischemic stroke during perioperative procedures

Mechanism	Content	Authors & Year
Thrombus shedding	Heart‐borne thrombosis sheds and reaches the brain with the blood flow	Maida CD,^12^ 2020
Brain local low perfusion	Chronic hypertension, diabetes, geriatric atherosclerosis, and other factors leading to vascular stenosis	Campbell BC,^13^ 2019 Kam PCA,^14^ 1997
	Low blood pressure and slow blood flow during surgery for a long time	Bijker JB,^15^ 2013
	Surgical trauma or tissue damage causing increased blood viscosity	
Sudden fluctuations in blood pressure	Surgery and anesthetic stimulation causing blood pressure to rise or drop sharply	Anadani M,^16^ 2021
Embolism	Fat, air, or cancer embolism	Keller K,^17^ 2020

## 2 MORBIDITY AND MORTALITY

A recent retrospective analysis, including 370,000 perioperative stroke patients, found the incidence of ischemic stroke to be 0.7% after a partial colectomy, 0.2% after a total hip replacement (0.2%), and 0.6% after a pulmonary surgery, 2.2%–5.2% after neurosurgery, and up to 2%–10% after cardiac and microvascular surgery.^18^ The risk of PAIS in the elderly population increases with age,^19^ from 0.1%–0.2% under 65 years of age to 0.5% between 65 and 74 years of age, and 1.0% over 75 years of age.^20^, ^21^ Despite current improvements in surgical techniques and surgical treatments, the incidence of perioperative strokes has not decreased significantly, increasing to 0.8% in patients undergoing non‐cardiac major vascular surgery. The mortality rate of perioperative strokes is 18%–26% higher compared with non‐operative stroke patients.^22^

## 3 RISK FACTORS

Perioperative stroke is associated with multiple risk factors, as detailed in Table 2

**TABLE 2:** Risk factors for perioperative stroke

Preoperative	During surgery	After surgery
Age >70^23^, ^24^	Type and nature of the surgery^14^	Heart failure
Female^25^, ^26^	Duration of the operation	Myocardial infarction
History of ischemic stroke or TIA Combined with other system diseases	Atherosclerotic lesions of The proximal aorta	Arrhythmia Atrial fibrillation^27^, ^28^
Hypertension	Anesthesia methods and management	Dehydration
Diabetes	General anesthesia	Blood loss
Coronary heart disease^29^	Local anesthesia	Long‐term bedridden
Chronic obstructive^17^	Liquid restrictions	Hyperglycemia
Pulmonary disease	Arrhythmia	Low blood fraction
Renal insufficiency	Hyperglycemia	
Carotid artery stenosis^30^, ^31^	Hypotension	
Ascending aorta sclerosis	Hypertension	
Stop antithrombotic therapy suddenly^32^		

## 4 PATHOPHYSIOLOGY OF PERIOPERATIVE STROKES

Perioperative stroke is dominated by ischemia and embolism. In an ischemic stroke, vascular occlusion leads to the disorders of local blood supply in the corresponding brain regions,^33^ which induces a complex series of cascade reactions at the (sub)cellular and molecular levels,^34^, ^35^ and ultimately leads to cellular and tissue damage.^36^, ^37^, ^38^ The pathological biochemical reaction of an ischemic stroke begins with energy deprivation induced by a lack of oxygen and glucose supply to local brain tissue.^39^, ^40^ This is followed by stimulation of neuronal depolarization and glutamate release, causing calcium inward flow and elevated sodium ion content in the intracytoplasmic, and more glutamate release which leads to cellular excitotoxicity and cellular swelling,^41^ ion channel dysfunction, and massive reactive oxygen and/or nitrogen species, ROS/RNS or RONS production.^42^

These pathological and biochemical changes at the cellular and molecular levels further spread to neighboring cells, activating a series of enzymatic cascade reactions that eventually lead to the cell membrane and mitochondrial damage and production of RONS^43^, ^44^; the production of RONS can further damage mitochondria and DNA, eventually leading to cellular necrosis or apoptosis.^45^, ^46^, ^47^, ^48^ Inflammatory mediators or cytokines secreted by necrotic or apoptotic cells activate resting microglia in the brain and promote the invasion and infiltration of peripheral neutrophils and macrophages^49^, ^50^; activated microglia in the brain can further converge and aggregate toward damaged neurons,^51^ mediating the release of pro‐inflammatory factors, and start a vicious circle, aggravating neuronal damage.^52^, ^53^, ^54^, ^55^

## 5 POTENTIAL NANOTHERAPEUTIC STRATEGIES FOR PERIOPERATIVE STROKE

Nanotechnology is the science and technology of making substances from individual atoms and molecules, and it studies the properties and applications of materials with structural dimensions in the range of 1–100 nanometers.^56^, ^57^ Nanotechnology has developed rapidly in the last few decades and shows potential in the diagnosis and treatment of diseases.^58^, ^59^, ^60^ The properties of nanomaterials differ significantly from those of equivalent materials at the corresponding macroscopic scales due to the different arrangement and spacing of surface atoms and molecules.^61^

Nanomaterials have a great potential for biomarker development, disease diagnosis, and disease treatment, which are as follows: targeting damaged cells or tissues through molecular‐scale interactions with improved and modified nanomaterials^62^, ^63^; controlled release of drugs by nano‐engineered materials,^64^, ^65^, ^66^ improving bioavailability,^67^, ^68^ transporting multiple drug formulations, and protecting drug compounds from degradation through different molecular modifications on the surface.^69^ Nanomaterials are also a good alternative for developing drug strategies to penetrate the blood–brain barrier (BBB) by surface‐functionalized ligand modifications that target and penetrate the BBB and increase its half‐life in the blood circulation.^70^, ^71^ The passive/active targeting properties and improvement of the biostability of neurotherapeutic drugs increase the drug concentration in the pathological injury zone to achieve the desired therapeutic effect.^72^, ^73^, ^74^ Nanotechnology provides a convenient platform for immobilizing and loading specific molecules or drugs on nanocarriers at higher loading rates. Nanomedicines also possess neuroprotective effects.^75^, ^76^, ^77^ These properties of nanomaterials place them at the forefront of future precision diagnosis and treatment of central nervous system diseases, such as ischemic stroke.^78^

Nanodrug delivery systems have unique advantages in the treatment of ischemic stroke, including enhanced BBB permeability,^79^, ^80^ targeting, and modulating drug release.^81^ Most studies on nanotechnology‐based therapies for ischemic stroke have focused on targeting revascularization, antioxidative stress, inflammation, and apoptosis, and promoting neuronal regeneration^82^ as shown in Table 3.

**TABLE 3:** Potential nanotherapeutic strategies for perioperative stroke

Nanotherapeutic strategies	Carrier type/materials	Drug(s) delivered	Major findings and comments	Authors & Year
Revascularization	Antioxidant nanoparticles	t‐PA	Extended the in vivo half‐life of t‐PA in systemic circulation, improved its bioavailability, and extended therapeutic window	Mei T,^83^ 2019
	Soft discoidal polymeric	t‐PA	Preserved lytic activity, the deformability, and blood circulating time, together with the faster blood clot dissolution	Colasuonno M,^84^ 2018
	gold@mesoporous silica core–shell nanospheres	uPA	A near‐infrared‐triggered controlled release on demand, hyperthermia‐enhanced thrombolysis locally for decreasing drug dosage	Wang X,^85^ 2017
	Mesoporous/macroporous silica (MMS)/platinum (Pt) nanomotor (MMNM) coated with platelet membrane (PM)	Urokinase/Hep	The motility of the nanomotor can effectively facilitate its deep penetration into the thrombus site and improve retention	Wan M,^86^ 2020
	Polymeric nanoparticles wrapped with membranes platelet membrane cloaked polymeric nanoparticles (PNP‐PA)	Alendronate sodium	Enhanced drug accumulation at skeletal sites and reduced off‐target effects	Matrali SSH,^87^ 2020
		t‐PA	PNP‐PA exhibited potent innate targeting and local clot degradation with a low risk of bleeding complications	Xu J,^88^ 2020
Scavenging reactive oxygen species	CeO_2_ nanoparticles	ZIF−8	Exhibits prolonged blood circulation time, reduced clearance rate, improved BBB penetration ability, and enhanced brain accumulation	He L,^89^ 2020
	Framework nucleic acid	Anti‐C5a aptamers	Selectively reduce C5a‐mediated neurotoxicity and effectively alleviate oxidative stress in the brain	Li S,^90^ 2019
	Polyoxometalate nanoclusters	Mo	Excellent scavenging activity of ROS by changing their reduced and oxidized status	Li S,^91^ 2019
Anti‐inflammation	Liposomal	9‐aminoacridine (9‐AA)	Liposomal 9‐AA can efficiently encapsulate 9‐AA, exhibit anti‐inflammatory activities through an NR4A1/IL−10/SOCS3 signaling pathway and modulate the microglia activation	Wang H,^92^ 2020
	ROS‐responsive and fibrin‐binding polymers micelle	Rapamycin	The combination of micelle facilitated ROS elimination and anti‐stress signaling pathway interference under ischemia conditions	Lu Y,^93^ 2019
	Platelet‐mimetic nanoparticles (PTNPs) co‐loaded with piceatannol	Piceatannol	Decrease neutrophil infiltration and reduce infarct size, monitor the inflammatory neutrophils coupled with magnetic resonance imaging	Tang C,^94^ 2019
Neuronal regeneration	Degradable nanomaterials	CAT/SOD	Provide a good microenvironment for neural progenitor cell activation and migration after cerebral infarction and promote endogenous neuronal regeneration	Petro M,^95^ 2016
	Magnetosome‐like ferrimagnetic iron oxide nanochains (MFIONs)	DNA/PEI	Ferrimagnetic nanochains‐based mesenchymal stem cell engineering augment the homing ability of the engineered MSCs to the ischemic cerebrum for highly efficient post‐stroke recovery	Zhang T,^96^ 2019
	Magnetic nano‐vesicles (MNV)	Iron oxide nanoparticles (IONP)	Promoted the anti‐inflammatory response, angiogenesis, and anti‐apoptosis in the ischemic brain lesion, thereby yielding a considerably decreased infarction volume and improved motor function	Kim HY,^97^ 2020

### 5.1 Nanodelivery strategies for revascularization

**FIGURE 1:**
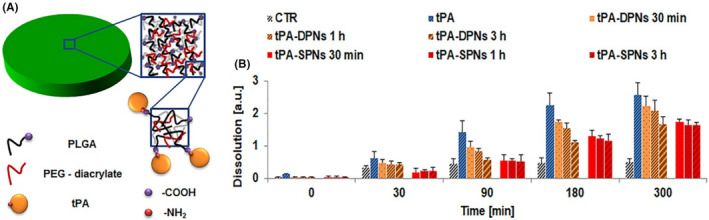
(A) Schematic representation of tPA‐DPNs, highlighting the porous structure of DPNs and their direct conjugation with tPA. (B) Dissolution of blood clots with tPA‐DPNs and tPA‐SPNs, pre‐incubated with FBS for 30 min, 1 h, and 3 h. A direct comparison is provided with fresh, free tPA [*n* = 10]. Reprinted (adapted) with permission from Ref. [^84^]. Copyright 2018 American Chemical Society

Currently, tissue plasminogen activator (tPA) intravenous thrombolysis remains the standard clinical treatment for patients with acute ischemic stroke within 4–5 h after the onset of ischemic stroke.^98^, ^99^ However, due to the limited "therapeutic window," only a minority of patients (<10%) receive this treatment. To broaden the therapeutic window of tPA, Mei et al.^83^ designed a t‐PA‐installed, nitroxide radical‐containing, self‐assembled polyion complex nanoparticles (t‐PA@iRNP). This improved pharmacological performance of t‐PA@iRNP prevented the rapid metabolism and excretion out of the body after systemic circulation, thus remarkably extending the in vivo half‐life of t‐PA. Marianna et al.^84^ constructed a nanotherapeutic agent by directly combining the clinical formulation of tPA with the porous structure of soft discoidal polymer nanostructures (TPA‐DPNs) (Figure 1A). Due to the protective effect of the porous matrix in DPNs, tPA degrades slowly in serum, and TPA‐DPNs retained more than 70% of its original activity after 3 h of exposure to serum protein (Figure 1B).

**FIGURE 2:**
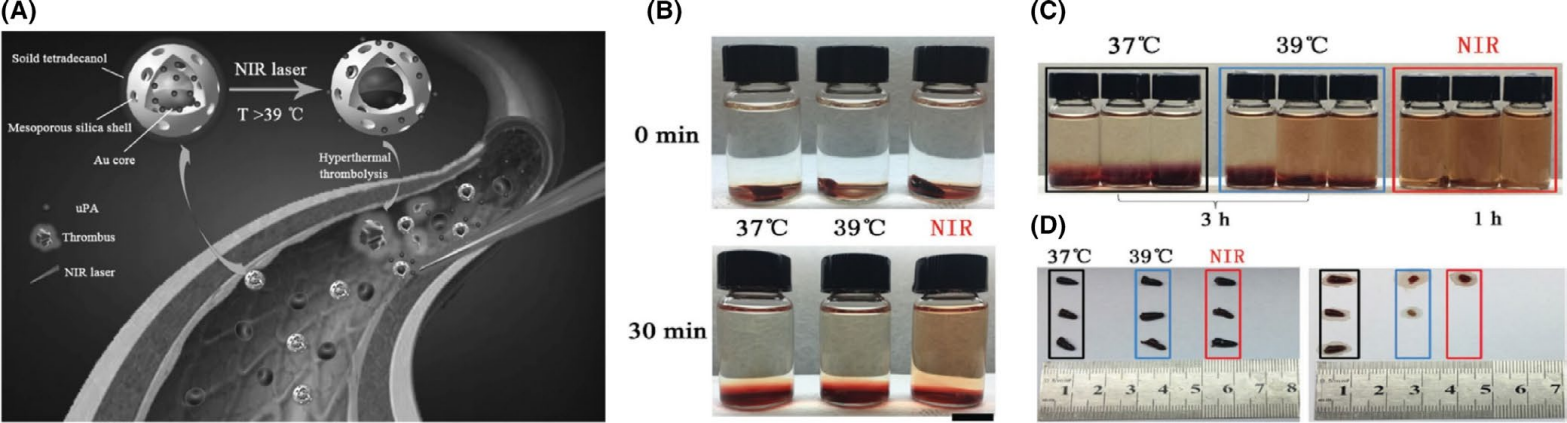
(A) Schematic illustration of the fabricated uPA controlled release system and enhanced thrombolysis *in vivo*. (B) Photos of thrombolysis in saline containing 200 µg ml^−1^ uPA–NPs at 37°C, 39°C, and NIR irradiated temperature for 30 min, scale bar represents 0.5 cm; (C) Photos of thrombolysis in saline containing 200 µg ml^−1^ uPA–NPs at 37°C, 39°C for 3 h and irradiation of NIR for 1 h; (D) Photos of thrombus before and after treated at 37°C (black frame), 39°C (blue frame) and NIR (red frame). Reprinted (adapted) with permission from Ref. [^85^]. Copyright 2017 John Wiley and Sons

Ischemic stroke slows the blood flow by vascular obstruction and higher doses of tPA are needed for effective thrombolytic therapy, which may result in cerebral edema and cerebral hemorrhage. To improve the thrombolytic effect, Wang et al.^85^ designed a nanodelivery system based on gold@mesoporous silica core‐shell nanospheres (Au@MSNs) (Figure 2A), which uses a near‐infrared (NIR) laser (808 nm) to trigger the release of tPA. tPA is encapsulated with the phase change material 1‐tetradecanol (Tet) into the pore of Au@MSNs. Laser irradiation is expected to release tPA from the nanocarrier when 1‐tetradecanol is reconverted to liquid due to the photothermal conversion of gold (Figure 2B). The photothermal‐only treatment group also has a thrombolytic effect (Figure 2C,D). The tPA‐NPs achieved targeted release of tPA and enhanced the thermotherapeutic thrombolytic effect locally on NIR laser irradiation. Further, ultrasound energy enhanced the efficacy of thrombolytic drugs. Daffertshofer et al.^100^ showed that ultrasonic waves at 300‐KHZ penetrated the bone efficiently and exposed the entire brain to ultrasound. However, there was a 36% hemorrhage rate in the group treated with rt‐PA plus ultrasound. Focused ultrasound of the Willis circle, with or without microbubbles, appears to be a promising means of improving the efficacy of intravenous thrombolytic drugs. A larger phase III trial is currently being tested.^101^

Tissue plasminogen activator activation of fibrinolytic is systemic and does not target specific regions for fibrin activation. To improve the accuracy of tPA thrombolysis, Wan et al.^86^ designed a mesoporous/macroporous silica (MMS)/platinum (Pt) nanomotor (MMNM) coated with platelet membrane (PM) (called MMNM/PM). The large‐sized thrombolytic drug urokinase was loaded into the nanomotor's macroporous structure and the anticoagulant drug Hep was loaded into the mesoporous structure. Regulated by a special protein on the PM, the nanomotor targeted the thrombus site, and then, the PM was fractured on NIR irradiation and rapidly released thrombolytic urokinase (3 h) and sustained release of anticoagulant heparin (>20 days), sequentially releasing two drugs. Meanwhile, the motility of the nanomotor under NIR irradiation effectively facilitated its deep penetration into the thrombus site and improved retention. Hu et al.^87^ also constructed a nanocarrier with a bone‐targeted core–shell structure in which platelet membranes were wrapped around the surface of polymeric nanoparticles. Alendronate sodium (Ald) is used as a targeted ligand for its ability to chelate calcium ions in the bone microenvironment, enhancing drug accumulation at the skeletal site and reducing off‐target effects. Xu et Al.^102^ developed platelet membrane cloaked polymeric nanoparticles (PNP) conjugated with rt‐PA on the surface to achieve clotting targeting thrombolytic therapy. PTP‐PA exhibited strong innate targeting and local clot degradation after intravenous administration in different animal models of thrombus, including mesenteric artery embolism, PE, and ischemic stroke, indicating the excellent therapeutic potential in a broad spectrum of thrombo‐related diseases. These innovative nanotechnologies open up new avenues for tPA‐based stroke therapy.

### 5.2 Nanodelivery strategies for scavenging reactive oxygen species

Recent advances in nanomedicine have facilitated the development of functional nanomaterials, such as carbon, vanadium, manganese, platinum, and cerium nanoparticles with higher antioxidant activity and stability than natural enzymes.^103^, ^104^ Liu et al.^75^ revealed the detailed mechanism of the antioxidant effect of water‐soluble MeNPs by comprehensively analyzing their scavenging activity against a variety of RONS. The results showed that PEG‐MeNPs had a catalytic mechanism for O_2_‐ that mimicked SOD. Compared with natural antioxidant enzymes (e.g. SOD), the nano‐antioxidants targeted specific RONS, PEG‐MeNPs and exhibited a wide range of antioxidant activities against a variety of toxic RONS, including ‐OH, O_2_‐, H_2_O_2_, ‐NO, and ONOO‐, highlighting the strong scavenging capacity of reactive oxygen species by nano‐antioxidants. In vitro experiments showed that PEG‐MeNPs have neuroprotective and anti‐inflammatory effects. In vivo results further demonstrated that MeNPs have a unique combination of antioxidant, anti‐inflammatory, and biocompatibility, which effectively protects against ischemic brain injury with negligible side effects. A new strategy for the in situ synthesis of ZIF‐8‐covered CeO_2_ NPs (CeO_2_@ZIF‐8) was explored by He et al.^89^ The surface ZIF‐8 acted as a peroxidase that maintained antioxidant activity in the presence of excess H2O2 or other oxidants and absorbed H_2_O_2_ and broke O─O bonds to decompose H_2_O_2_ with enhanced catalytic and antioxidant activities.

**FIGURE 3:**
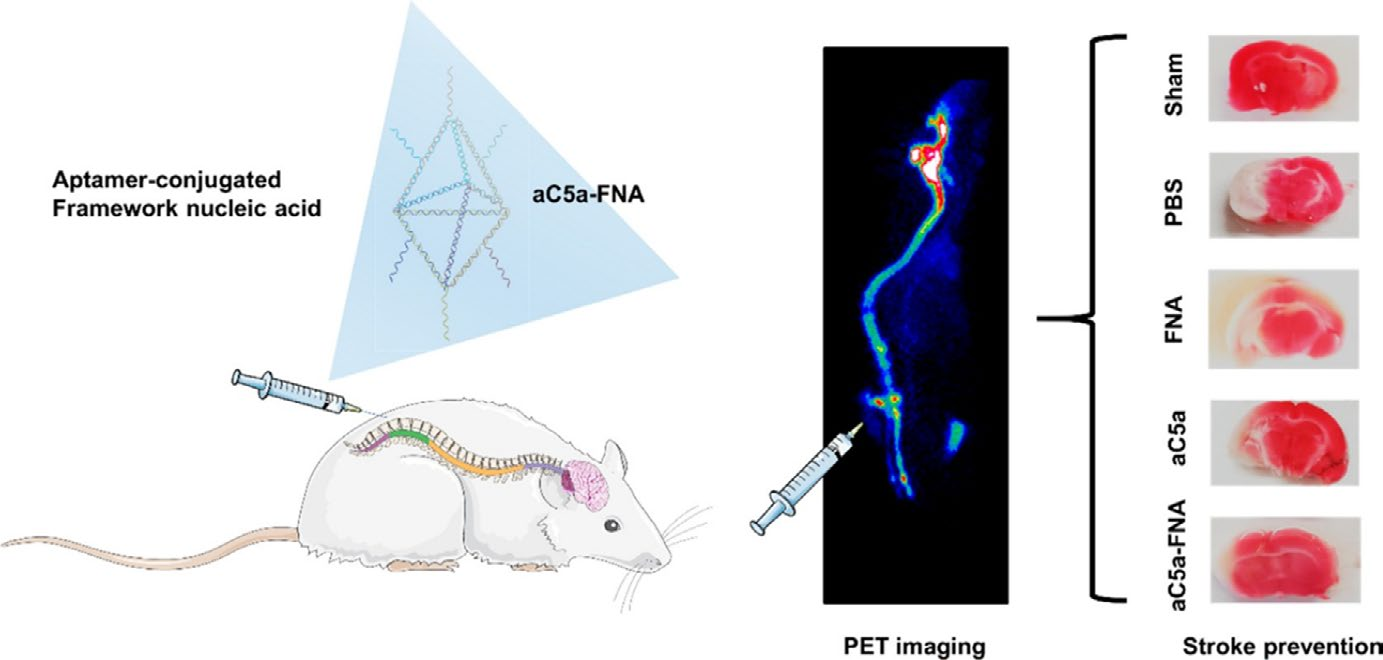
Schematic of cerebral ischemia‐reperfusion injury (IRI) treatment using an anticomplement component 5a (aC5a) loaded framework nucleic acid (aC5a‐FNA) after intrathecal injection. PET imaging and brain tissue staining confirmed the biodistribution and treatment efficacy of FNA for brain IRI management. Reprinted (adapted) with permission from Ref. [^90^]. Copyright 2019 American Chemical Society

Furthermore, it has been shown that ischemic stroke triggers activation of the complement system, leading to an increase in aC5a in plasma and ischemic penumbra, triggering an inflammatory cascade response and exacerbating the vicious cycle between oxidative stress and inflammatory responses. Li et al.^90^ prepared a bipyramidal FNA loaded with C5a aptamers (aC5a‐FNA) (Figure 3). After intrathecal injection, ac5A‐FNA selectively alleviated C5A‐mediated neurotoxicity and effectively relieved oxidative stress in the brain. Another study^91^ also showed that ultra‐small molybdenum polyoxomethoic acid nanoclusters (POM) had excellent ROS scavenging capability by changing their reduction and oxidation states. After intrathecal injection, POM nanoclusters were preferentially uptake by the brain, leading to rapid accumulation of POM nanoclusters in the ischemic penumbra, alleviating oxidative stress and inflammatory injury, effectively inhibiting neuronal apoptosis after brain I/R injury, and restoring neuronal function.

### 5.3 Nanodelivery strategies for anti‐inflammation

The acute systemic inflammation induced by surgery induces or exacerbates ischemic brain injury. The inflammatory response has important implications for stroke susceptibility and prognosis and is involved in the pathophysiological process of stroke. During this process, TNF‐α, IL‐1, IL‐6, and C‐reactive protein levels are significantly increased. They activate microglia in the brain and stimulate invasive infiltration of peripheral leukocytes,^105^ thus accelerating ischemic damage and expanding the infarct area. Therefore, blocking the inflammatory response to alleviate injury is an extremely promising neuroprotective therapeutic strategy. Wang et al.^92^ found that 9‐AA can act as a novel NR4A1 activator to downregulate the activation levels of microglia and macrophages through the NR4A1/IL‐10/SOCS3 signaling pathway to mitigate inflammatory responses. However, the low therapeutic index and poor water solubility of 9‐AA greatly limit its application in vivo. To avoid the adverse effects of 9‐AA, they prepared a PEG/cRGD double‐modified liposome loaded with 9‐AA, which prolonged its blood circulation, and significantly reduced its side effects on the lung.

mTOR inhibitors have been reported to reduce neuroinflammation and reperfusion injury by polarizing microglia from pro‐inflammatory M1 type to anti‐inflammatory M2 type. In addition, inhibition of the mTOR pathway induced autophagy and removed damaged organelles to repair neurons. Lu et al.^93^ developed a polymer micellar system with neurovascular targeting and mTOR inhibition. Briefly, the mTOR inhibitor rapamycin was encapsulated in micelles formed by self‐assembly of fibrin‐binding peptide (CREKA) and reactive oxygen scavenging polymer (C‐PEg‐Lysb). In vivo results confirmed that the micelles enriched in ischemic sites and achieved the controlled release of drugs, induced the elimination of ROS, reshaped the phenotype of microglia, and alleviated neurovascular injury caused by oxidative stress; they also enhanced blood perfusion and neuroprotection.

**FIGURE 4:**
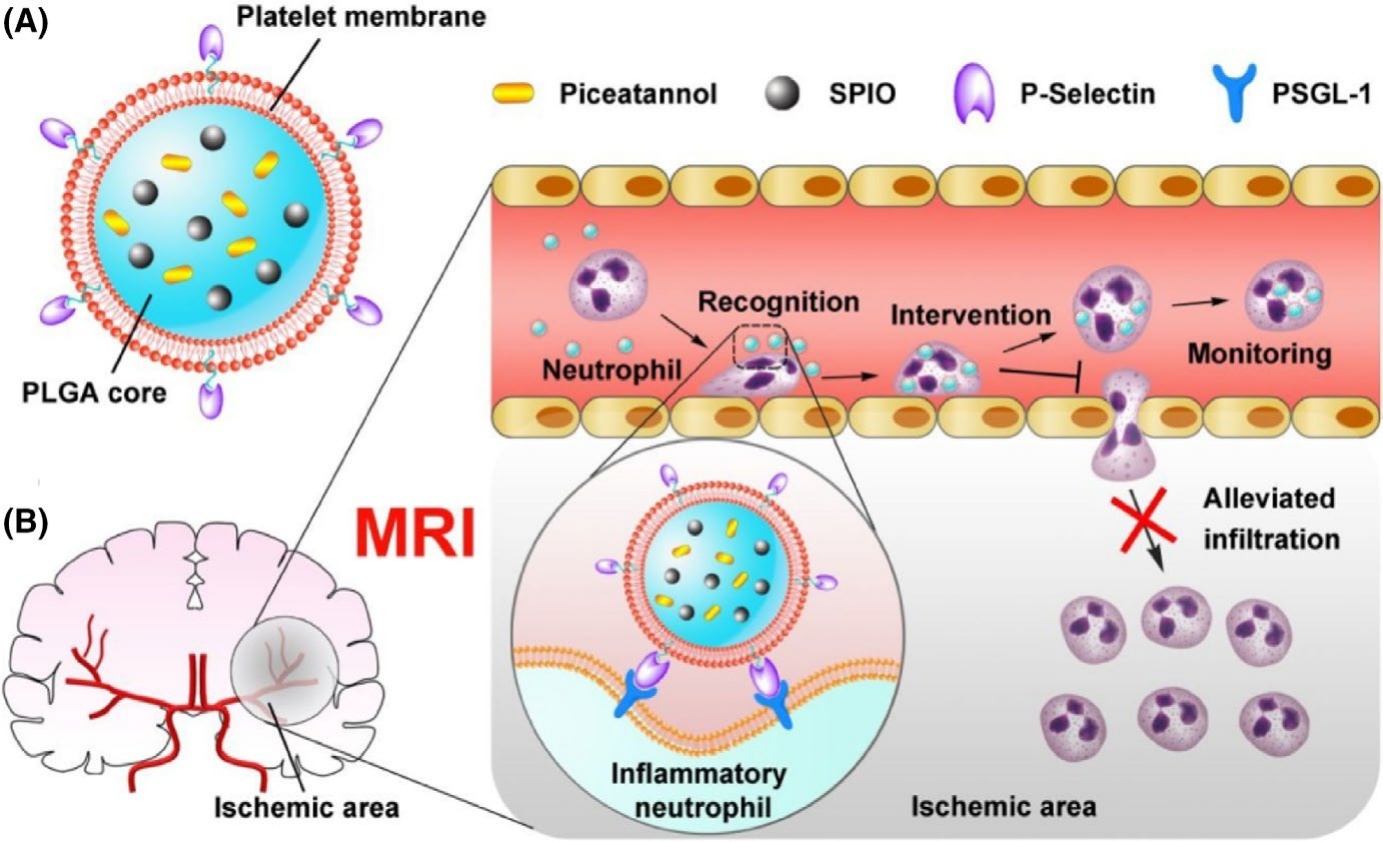
(A) A diagram of the main components of PTNPs. (B) Schematic of PTNPs reducing inflammatory neutrophil infiltration. Reprinted (adapted) with permission from Ref. [^94^]. Copyright 2019 American Chemical Society

Neutrophils autonomously migrate to the ischemic zone and release reactive oxygen species after receiving inflammatory signals during stroke, which is considered to be the main cause of reperfusion injury after AIS. Therefore, reducing inflammatory neutrophil infiltration may be an effective treatment for AIS. Based on the specific affinity between inflammatory neutrophils and activated platelets, Tang and other colleagues^94^ designed platelet‐mimetic nanoparticles (PTNPs) that directly identified, intervened, and monitored activated neutrophils during inflammation (Figure 4A). After intravenous injection, PTNPs selectively recognize inflammatory neutrophils *via* a specific affinity between p‐selectin and the pskl‐1 microregion. PTNPs were then internalized into adherent neutrophils where the loaded piceatannol was released, thus promoting the detachment of neutrophils from endothelial cells into circulation, resulting in decreased neutrophil infiltration (Figure 4B). In vivo results showed that this bionic strategy reduced inflammatory infiltration of neutrophils, decreased infarct volume, and improved the neurological function of ischemic stroke.

### 5.4 Nanodelivery strategies for neuronal regeneration

Ischemic stroke can present with impairment or loss of neuronal regenerative capacity, progressively causing impaired neuronal function or structural changes. Thus, improving neuronal regenerative capacity has potential in the treatment of ischemic stroke. Petro, M and other colleagues^106^ injected tPA into the carotid artery 3 h after ischemic stroke for post‐ischemic reperfusion, followed by immediate injection of degradable nanomaterials (nano‐CAT/SOD) encapsulated with antioxidants CAT and SOD. The results showed that the nano‐CAT/SOD group presented more neuronal cells in the brain of ischemic stroke rats, and neuronal cells migrated from the subventricular layer of the lateral ventricle into the anastomotic lateral flow, and nano‐CAT/SOD reduced neutrophil infiltration in the brain and inhibited neuronal cell apoptosis. This suggests that nano‐CAT/SOD may provide a good microenvironment for neural progenitor cell activation and migration after cerebral infarction and promote endogenous neuronal regeneration. Some growth factors such as erythropoietin, epidermal growth factor, nerve growth factor, and brain‐derived neurotrophic factor have been found to stimulate neuronal regeneration. Zhang et al.^96^ designed multimeric nanoparticles containing epidermal growth factor (to stimulate neural stem/progenitor cell proliferation) or erythropoietin (to reduce neonatal apoptosis).

**FIGURE 5:**
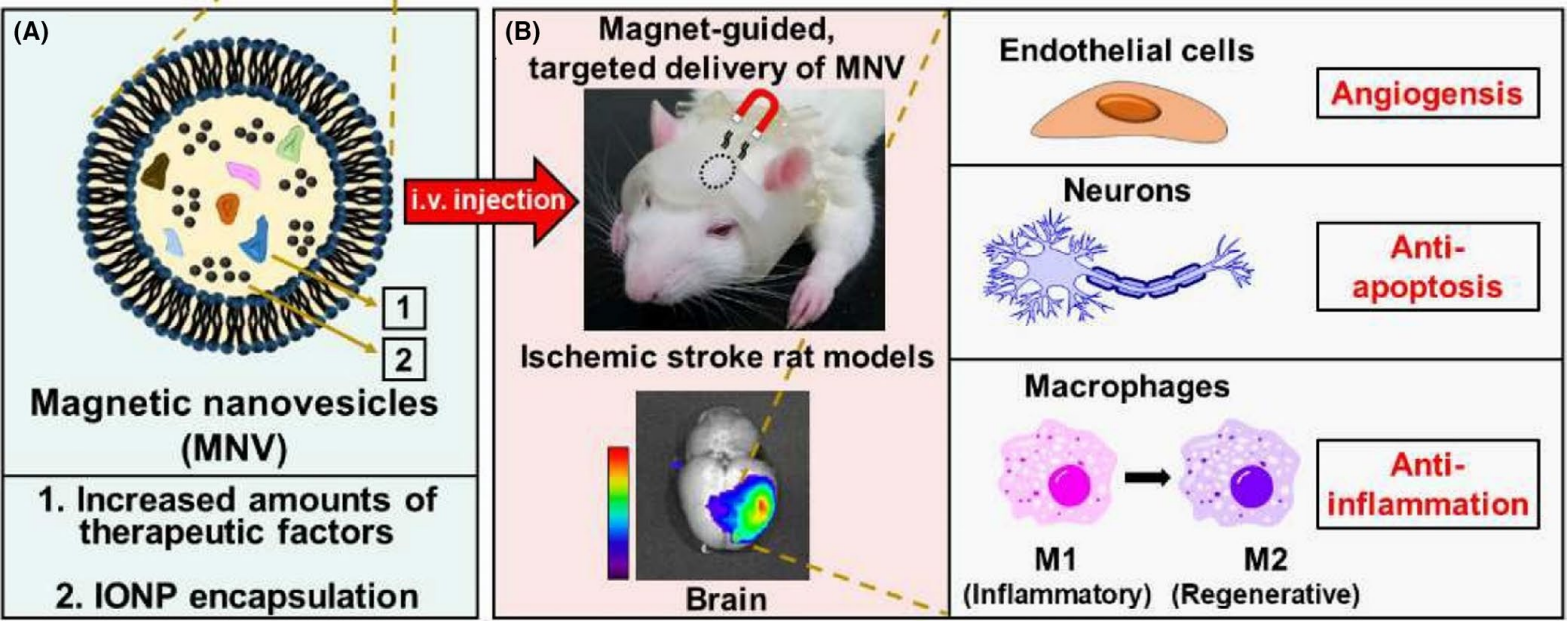
(A) MNV loaded with increased amounts of therapeutic factors and IONP. (B) Magnet‐guided delivery of MNV to an ischemic stroke lesion in magnetic‐helmet‐wearing rats and therapeutic effects of the MNV on various cells in the ischemic lesion. Reprinted (adapted) with permission from Ref. [^97^]. Copyright 2020 Elsevier

In addition, Han^97^ demonstrated that magnetic NV (MNV) derived from MSCs containing iron oxide nanoparticles (IONP) improved targeting and therapeutic efficacy to ischemic lesions (Figure 5). Because IONP stimulates the expression of therapeutic growth factors in MSCs, MNV contains more of these therapeutic molecules compared to NV derived from innocent MSCs. In a transient middle cerebral artery occlusion (MCAO) rat model, the magnetic navigation increased the localization of MNV to ischemic lesions by 5.1‐fold after systemic injection of MNV. Injection of MNV and magnetic navigation promoted anti‐inflammatory responses, angiogenesis, and anti‐apoptosis in ischemic brain lesions, resulting in a significant reduction in infarct volume, and improved motor function.

In conclusion, the ideal stroke treatment should be beneficial in antagonizing both primary and secondary neuronal damage caused by an ischemic stroke. Theoretically, combined therapies that alleviate early ischemic–hypoxic injury may provide durable neuronal protection, and promote neuronal regeneration in ischemic stroke, and may be beneficial in expanding the therapeutic time window, minimizing drug side effects, and maximizing the intensity of the ischemia/reperfusion‐induced neuronal injury.

## 6 FUTURE PERSPECTIVES

In the last decades, nanotechnology has made great contributions to the treatment of ischemic stroke. Compared with traditional treatment methods, nano‐formulations and nanodrug delivery systems have significant advantages in improving drug penetration into the BBB, increasing drug bioavailability, and maintaining and controlling drug release. However, as an emerging technology, safety issues such as the biocompatibility of nanotechnology still exist, and technical improvements are still needed. Most of the nanotechnology‐based ischemic stroke diagnostic and therapeutic applications are still at the preclinical stage, and further clinical data are lacking to facilitate their clinical translation. This review focuses on the bioavailability, biosafety, biodegradation, and specific targeting of nanomedicines and nanodrug delivery systems, which is the key to the translation of nanomedicines from bench to bedside. It is necessary to optimize the size, structure, function, physicochemical properties and other parameters of nanoparticles to make them efficient nanomedicines or drug carriers with low side effects. Soon, nanotechnology may become an effective diagnostic and therapeutic tool for ischemic stroke, and even both. With the rapid development of nanomedicine and in‐depth study of stroke mechanisms, nanotechnology will play an important role in the clinical application of stroke.

## CONFLICTS OF INTEREST

The authors declare that the research was conducted in the absence of any commercial or financial relationships that could be construed as a potential conflict of interest.

## AUTHOR CONTRIBUTION

JYA drafted the manuscript. RRD and KS searched for relevant literature and were responsible for the major revisions. JJS and JJL revised the manuscript critically. LZ and WXL were involved in preparing the figures. JLY and YL were involved in preparing the tables. LL and ZZZ provided professional guidance for this review and performed a final check of the manuscript. All authors contributed to the review of this manuscript and approved the submitted version.

## 7 DATA AVAILABILITY STATEMENT

All data needed to evaluate the conclusions in the paper are present in the paper. Additional data related to this paper may be requested from the authors.
